# Learning the ABCs of pregnancy and newborn care through mobile technology

**DOI:** 10.3402/gha.v8.29340

**Published:** 2015-12-14

**Authors:** Angela Afua Entsieh, Maria Emmelin, Karen Odberg Pettersson

**Affiliations:** Division of Social Medicine and Global Health, Department of Clinical and Social Sciences, Faculty of Medicine, Lund University, Malmo, Sweden

**Keywords:** mHealth, mobile technology, pregnancy, newborn care, maternal health, content analysis

## Abstract

**Background:**

The diffusion of mobile phones in low- and middle-income countries has taken place faster than any other infrastructural development. Mobile Midwife, a mobile application implemented in Ghana in 2010, sends timely messages in local languages to registered expectant mothers and new parents. The field of mobile health (mHealth) is severely underresearched, yet it can be an alternative for improving health systems and the ways in which health services are delivered.

**Objective:**

Our goal was to investigate the role that Mobile Midwife technology has played in the lives of pregnant and nursing mothers in Awutu Senya District, Ghana.

**Design:**

A total of three focus group discussions and 19 individual interviews were conducted. Discussions and interviews were recorded, transcribed verbatim from the local language to English, and analyzed by means of qualitative content analysis at the manifest and latent levels.

**Results:**

The main findings show that while oscillating between modern and traditional practices, women gradually gained trust in Mobile Midwife's counselling and attempted to balance between myths and reality regarding nutrition in pregnancy. In addition, their decisions to seek essential obstetric care were enhanced by Mobile Midwife's advice. Women also felt strengthened in their understanding of the importance of seeking professional care during pregnancy and childbirth as well as recognizing signs of ill health in the newborn.

**Conclusions:**

The findings indicate that Mobile Midwife could be an excellent tool in working towards the improvement of maternal health. Mobile Midwife will hopefully contribute to the stepwise achievement of the Sustainable Development Goals extended from the Millennium Development Goals, which expire at the end of 2015. There is a need for strong political will from key stakeholders, to embark in the field of mHealth as a complementary means to strengthen health systems.

*Mobile health* (mHealth), a segment of eHealth (electronic health), is defined as ‘medical and public health practice by mobile devices such as mobile phones, patient monitoring devices, personal digital assistants (PDAs), and other wireless devices’ (1). The number of mobile phone subscribers worldwide is approximately 5 billion (1), and the diffusion of mobile phones in low- and middle-income countries is happening faster than any other infrastructural development (1–3). Various leaders in low- and middle-income countries have shown interest in mobile technology interventions as complementary approaches to delivering health care information (1).

According to Ronsmans and Graham (4), maternal health is one of the most important indicators of the success of a country's health system, and maternal mortality rates are still predominantly high in sub-Saharan Africa and southern Asia. The Safe Motherhood Initiative is a framework to tackle and improve maternal health globally (5) and the target for the fifth Millennium Development Goal (MDG) is to reduce maternal mortality by 75% between 1990 and 2015. Considering these goals it may be especially pertinent to consider relevant ways to tackle maternal morbidity and mortality in resource-poor settings. Lund et al. (6) claim that maternal health interventions involving mobile phone usage increased access to antenatal care (ANC) services in Zanzibar. This finding may indicate that mHealth interventions in low-income settings would be one important means to improve health outcomes (2, 7, 8).

Few studies were found to have investigated the benefits of health interventions involving the use of mobile phones. In a study by Jennings et al. (9), the ‘perceived’ benefit was that mobile phones were useful for receiving information and reminders concerning adherence to medication. Another study (10) reported an increase in facility-based births in Rwanda from 72 to 92% before and after the SMS-based mobile intervention, respectively. Similarly, Lund et al. (11) reported that use of SMS-based mobile phone interventions in Tanzania significantly increased skilled delivery attendance by 60% in the intervention group as compared to 47% in the control group. Likewise, a mobile phone intervention in Thailand improved the percentage of women and children who were included in ANC and in an expanded program on immunization, thus reducing delays in both antenatal visits and immunizations (12).

## The Ghanaian context

Mobile Technology for Community Health (MoTeCH) is an mHealth initiative introduced in Ghana in response to challenges experienced by rural communities (13–16). MoTeCH has two components: Mobile Midwife and Nurse Application, and this study focuses on the former. Mobile Midwife is a mobile application that sends timely messages in local languages to specified clients, mostly expectant mothers and new parents. The messages give relevant information concerning health and notify the client in instances of a missed appointment (8, 13). Currently the MoTeCH project in Ghana covers four out of a total of ten regions: the Upper West, Central, Greater Accra, and Volta Regions. MoTeCH was first implemented in the Upper East Region of Ghana, in 2010. Coincidentally, free maternal health care services were also introduced in four regions, including the Upper East Region and Central Region (study setting). These regions were rated among the poorest regions, recording the lowest levels in use of ANC services (14, 15).

## Health belief model

The health belief model (HBM) as explained by Nutbeam and Harris (17) is a model that was originally designed to understand the choice to participate in immunization or cancer screening programs but was later expanded to understand long-term behavioral change. It is now also used to predict health-related behaviors. It will further be used to understand how Mobile Midwife influenced the study participants.

The model has four theoretical constructs: perceived susceptibility (subjective assessment of the risk of developing a problem), perceived seriousness (subjective assessment of how severe the problem could be and its related consequences), perceived benefits (subjective assessment of the advantages of having taken action or initiated change), and perceived barriers (subjective assessment of the possible factors that would prevent one from initiating a behavioral change). Self-efficacy, a core element of the HBM, relates to the self-confidence and perceived ability of the individual in order to achieve a behavioral change (17).

Among regions of low- and middle-income countries, the African region has the lowest activity in mHealth initiatives (1) and the majority are still in their pilot phases. There have been very few evaluations of the possible benefits of introducing mHealth initiatives to improve and increase access to maternal health services in these regions. This lack of evidence for the efficacy of mHealth innovations presents decision makers with little help when prioritizing mHealth innovations (1, 3, 16, 18).

This appears to be the case in Ghana, where the Mobile Midwife program was implemented 5 years ago and has not yet been adequately evaluated. No study was identified that focused on how pregnant and nursing mothers experienced the Mobile Midwife program in Ghana. In order to provide feedback to the implementers and other key stakeholders, a qualitative study was considered pertinent. Knowledge regarding women's experiences of the intervention will assist in developing a quantitative instrument to measure the outcome and impact of the program. This is, therefore, the first study to qualitatively explore experiences of the Mobile Midwife program. The study aimed to investigate the role that the Mobile Midwife program has played in the lives of pregnant and nursing mothers in Awutu Senya District, Ghana.

## Methods

### Study design

A qualitative approach using semi-structured in-depth interviews (IDIs) and focus group discussions (FGDs) was employed (19). FGDs were included because they offer a good base for generating new knowledge and understanding of how the participants reason about the Mobile Midwife program (19–21). The IDIs were considered essential as they allow mutual interaction between researcher and informants (19). A thematic guide was used with open-ended questions organized around the topics: thoughts about Mobile Midwife and maternal and early parenthood experiences with Mobile Midwife. The method enabled both deeper understanding of topics in the interview guide raised by the researcher and discovery of new knowledge put forward by the informants (19, 22, 23).

### Study setting

Awutu Senya District is located in the Central Region of Ghana. The district has 3,078 surviving infants under 1 year of age and approximately 1,776 pregnant Mobile Midwife clients (24, 25). The district lacks a hospital and, therefore, Awutu Breku Health Centre in the district capital (Awutu Breku) functions as the main referral center for other health facilities in the region. Health service provision includes a mix of government, private, traditional, and spiritual practitioners (25). This district was selected because the Mobile Midwife program has been active here since its implementation in 2011.

### Study participants and data collection

FGD participants were selected according to their ownership of a mobile phone, and a mix of regular and non-regular antenatal clinic attendants was considered. Phone ownership classifications were as follows:

1. *Personal phone access* refers to those women owning a personal mobile phone.
2. *Household phone access* refers to women who used the phone of their spouse, friend, neighbor, or relatives (13).

In this study, the term *nursing mother* was used to refer to a mother with an infant less than or equal to 1 year of age.

Participants were purposefully recruited (19) by community health workers for the FGDs when they made contact with the health facilities. The target was to have a study population with maximum variation, and the variations were reflected in the phone ownership classifications above. Up-to-date Mobile Midwife registry books were used to select participants. Apart from the selection criteria described above, participants were selected if they were active users of Mobile Midwife and had interacted with the program for at least a year from the time it was implemented, were pregnant, and/or had experienced Mobile Midwife in previous pregnancies (nursing mothers with babies 1 year old or less).

Participants that agreed to take part in the study were contacted via telephone by the principle researcher, AAE. Participants were recruited from three different communities within the district, to allow for a range of variation (19, 20). Awutu Ahentia, Awutu Bontrase, and Awutu Okwampa had 5, 12, and 8 participants, respectively. Since the participants were well acquainted with their respective health centers, it was more convenient for all (researcher, health workers, and participants) to use the health centers as the meeting place for the discussions. The discussions were conducted at the respective community health facilities on days when the health workers were not going out for community outreach and when the participants had no medical appointments. The participants, AAE (moderator), and the note taker (coordinator of the MoTeCH project in the district) sat in a circle and a relaxed atmosphere was allowed to prevail before the sessions began.

One of the focus groups had five participants and one had eight, which facilitated all participants expressing their views (18, 21). The other focus groups had 12 participants each, making interaction more difficult (22). The groups could, however, not be split due to logistical circumstances. FGD participants that seemed to have more stories to tell about the Mobile Midwife were asked if they were interested in the IDIs. Fourteen participants from the FGDs agreed to be interviewed in the IDIs; others declined due to busy schedules. Further, five respondents who did not participate in the FGDs were recruited through snowball sampling (19). These individuals were given the opportunity to meet the interviewer (AAE) and were given information about how the individual interviews were to be carried out, in order to create an atmosphere of trust and rapport (19, 20).

The FGDs and individual interviews varied from 30 to 60 min and were conducted in the local language, tape recorded, and transcribed verbatim from the local language to English by CAG and AAE. Validation of the translation was done by a local nurse-midwife. Data were collected over a 4-week period, February–March 2014. AAE analyzed the recently ended sessions in preparation for the next. Participants were recruited until saturation was achieved, that is, until no new information emerged (19).

### Analysis

Manifest and latent content analysis as explained by Graneheim and Lundman (26) were employed, which allowed for analysis of what the text actually said, giving rise to categories (manifest level) and themes (latent level). Transcribed manuscripts from FGDs and IDIs were read several times. Meaning units were identified and condensed, taking care not to lose their original meaning. The condensed text was analyzed line by line separately and as a whole, then appropriate codes were developed. The FGDs and IDIs were analyzed and coded separately by AAE and KOP. The codes from the FGDs and IDIs were subsequently printed and analyzed together by AAE. Similar codes reflecting the same aspect were then grouped to form categories. Disagreement and/or uncertainty regarding the label and content of any category or theme were discussed between all authors. Categories addressing various aspects of the same topic were later pooled together into themes by AAE, ME, and KOP (26).

### Ethical considerations

The Ghana Health Service Ethical Review Committee in Ghana approved the study. We followed the guidelines established according to the Helsinki Declaration and also by the Council for International Organizations of Medical Sciences (CIOMS) in collaboration with the World Health Organization (WHO), in protection of human subjects (27, 28). Consent to participate in the study and permission for the sessions to be recorded was obtained from the participants, who signed a consent form. Participants were made to understand that they were at liberty to cease taking part in the study without any resulting consequences. Focus group participants were reminded that there were going to be different experiences and opinions of Mobile Midwife utilization among them and that therefore there was no right or wrong answer and all should feel at liberty to share their views. The participants were also reminded that what was discussed in the groups was confidential and not to be discussed outside the groups.

## Results

**Fig. 1:**
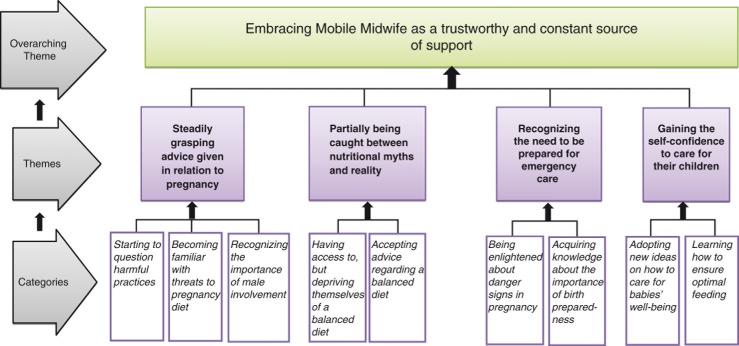
Benefits and experiences of the Mobile Midwife technology.

The study included a total of 29 participants: pregnant and nursing mothers in the age range of 20–35 years. They worked either as teachers, seamstresses, farmers, or traders, with education levels ranging from primary to senior high school and had interacted with Mobile Midwife between 1 and 3 years. The analysis yielded one overarching theme, *embracing Mobile Midwife as a trustworthy and constant source of support*, and four main themes: 1) *steadily grasping advice given in relation to pregnancy*, 2) *partially being caught between nutritional myths and reality*, 3) *recognizing the need to be prepared for specialized care*, and 4) *gaining self-confidence to care for their children*. The findings are presented below in the text and illustrated by quotes from the data. Themes are presented in bold and categories are further italicized. Figure 1 provides an overall picture of the analysis.

### Embracing Mobile Midwife as a trustworthy and constant source of support

*The ABCs of pregnancy and newborn care*, a concept derived from the analysis of our data, reflected practical and feasible advice, which recommended information specifically tailored towards meeting the needs of the pregnant and nursing mothers. *Embracing Mobile Midwife* refers to the process of adopting its new ideas and seeing it as a credible source of authority. As a trustworthy and constant source of support, Mobile Midwife gave the women a sense of direction and reassurance whenever they were faced with uncertainties during pregnancy and newborn care.

### Steadily grasping advice given in relation to pregnancy

*Steadily grasping advice* describes a slow but ongoing move by the pregnant and nursing mothers towards adopting new ideas from Mobile Midwife. The move towards change was not only going to affect the women, but also their relations with significant others like grandmothers and traditional birth attendants (TBAs). Letting go of old traditions passed on from generation to generation and adopting new ones did not come easily, as shown below. Nevertheless, in the course of time the women took the brave steps of adopting these new ideas and proving whether they were beneficial to them or not.

We thought that these people's [Mobile Midwife] ways are not good but this time we have seen that it is also good and has helped us. You see during the old times we did not understand these things about pregnancy so it was hard to change. (FDG 2)

#### Starting to question harmful practices

The older women, never having experienced health services during pregnancy, recalled unfortunate events giving birth at home attended by unskilled TBAs. Through Mobile Midwife, the women were beginning to understand the benefits of giving birth at the hospital.

… at the hospital when you are going to give birth and you have a complication, the nurses will be able to know what it is that is troubling you and they will take care of you and give you medication, unlike at home. (IDI-Inf. 11)

However, a concern expressed among the informants was influence from grandmothers and TBAs who adhered faithfully to harmful traditional practices.

They [untrained TBAs] also say that maybe if the child has an illness in the stomach or they say maybe the child is dirty, then when you douche the medicine will wash and cleanse the child so that is why I did it. (IDI-Inf. 4)

The message from Mobile Midwife was that they should be aware of dangers associated with use of enemas prior to labor. As Mobile Midwife's advice began to work to their own advantage, the women realized the value of attending the antenatal clinic and appreciated receiving appropriate medication since they were relying less on using local herbs.

They [Mobile Midwife] tell us that douching is not good because sometimes it interferes with the well-being of the baby. Sometimes douching [with very strong herbal mixtures] can even cause the pregnancy to terminate. (FGD 3)

#### Becoming familiar with threats to pregnancy diet

The women were unaware of the potentially negative effects of malaria on pregnancy and were enlightened about this aspect by the Mobile Midwife. Even though the informants had been entreated to sleep in mosquito nets, financial constraints made this difficult. Mobile Midwife's alternative suggestion of burning dry orange peels to repel mosquitoes was much appreciated and considered affordable. They appreciated that Mobile Midwife sent them reminders to go to the clinic when it was time to take the malaria prophylaxis medicine sulfadoxine/pyrimethamine (SP) – a procedure that improved their health.

There are some messages that I receive where they encourage me to go to the clinic and when I go there they give me SP every month. So I was very healthy. It is medicine that is given to pregnant women to prevent malaria. (IDI-Inf. 19)

#### Recognizing the importance of male involvement

A burden raised was the lack of domestic and financial support from their husbands during pregnancy. Having registered for Mobile Midwife with their husbands’ phones, the women disclosed that their husbands consequently were the first to receive the voice messages and then passed the phones to them. Gradually their husbands became interested in listening to the Mobile Midwife messages and often helped their pregnant wives as advised by Mobile Midwife.

Initially, even when I was pregnant I would pound *fufu* [mixture of yam and plantain], but during the time I was recently expecting, he would pound the *fufu* and even go to the farm and bring food home. (IDI-Inf. 3)

### Partially being caught between nutritional myths and reality

Food played an integral role in the daily lives of the women participating in this study. They belonged to a society where beliefs pertaining to foods to be avoided during pregnancy were a norm they were required to adhere to. Not practicing those beliefs could jeopardize both an unborn and infant's health physically as well as mentally, as indicated in the quote below.

They [grandmothers] discourage us from eating crabs because they say when the baby is born, he/she will be hard and stiff. (FGD 2)

However, being told by Mobile Midwife that some of these traditions were not only false but also posed a danger to them and their children left the women in a tug-of-war type of situation. Further still, ancient nutritional advice had allegedly been proven to harm no one, as shown below, so this gave the women no reason to want to believe the new ideas presented by Mobile Midwife.

I wasn't supposed to eat certain foods but I was forced to eat them … See our grandparents have been doing these things and nothing happened to them so that is why. (IDI-Inf. 5)

#### Having access to, but depriving themselves of a balanced diet

The women who were farmers and traders had access to a variety of foods and fruits, but nurtured their pregnancy mainly with foods made from fermented grain.

I used to have a lot of cravings for *moko* [pepper] and *banku* [fermented grain], I would just eat the *banku* with blended *moko* and onions. I would just eat like that without adding anything else and drink water, so with the previous [pregnancies] I was always weak like someone who has worked hard and is tired. (IDI-Inf. 3)

The informants also stayed away from eating certain fruits, a good source of vitamins in pregnancy due to their beliefs related to some fruits.

There is a belief that when you are pregnant and eat fruits like mango then the baby will always have a ‘running stomach’. (FGD 2)

#### Accepting advice regarding a balanced diet

Since most of the foods were readily available, Mobile Midwife was able to suggest a variety of food combinations that would serve as a balanced diet for the women. The informants voiced that after they started eating the recommended foods, their health improved and the fact that this was reflected in their medical reports increased their trust.

I received a message that advised me to eat *kontomere* [a green vegetable with blood-boosting abilities] and when I went to the lab I was told that my blood levels had improved, so that is what made me to believe in the messages I receive. (FGI 1)

### Recognizing the need to be prepared for emergency care

The expectant mothers lived in a community where more value was given to TBAs than health care professionals. Consequently, the custom of giving birth at home prevented the expectant mothers from seeking care from nurses and midwives. At the same time, their reliance on unskilled TBAs proved to be dangerous, as indicated below.

They [unskilled birth attendants] had been putting their hands in my vagina and doing all sorts of things for hours and I was very tired, I could not even push. They forced me to push and it even caused my private parts to rip open, I really suffered. (IDI-Inf. 4)

Interacting with Mobile Midwife increased the expectant mothers’ awareness of the importance of seeking skilled assistance from the health care centers, both during pregnancy and childbirth, particularly in case of complications.

As for the antenatal care, it helps. It's like if you don't … if you are pregnant and you do not visit the clinic every day or every month, it will trouble you because sometimes there are some medicines that are recommended by the doctors and nurses that you need to take. (IDI-Inf. 2)

#### Being enlightened about danger signs in pregnancy

The women misinterpreted some danger signs in pregnancy. For example they believed that swollen feet were a sign of a male child. Mobile Midwife provided proper explanations and encouraged them to seek care at the hospital.

Our grannies used to say when your feet get swollen it means you are about to give birth to a boy, but the Mobile Midwife says it is not like that … they say if you are pregnant and your feet swell we should be going to the clinic. (IDI-Inf. 19)

The participants were also suspicious and worried when varying signs of labor were experienced. Common signs mentioned were severe waist pains, backaches, frequent urinating, breaking of water, and constant contractions. Some of these signs were interpreted as a result of the ‘evil eye’, which led them to seek help from spiritualists, but Mobile Midwife encouraged them to not be alarmed as it was part of normal pregnancy. In case of bleeding, persistent headache, or dizziness they were requested to seek urgent assistance.

When it was 3 months left they sent me a message saying when I notice that I have terrible headache or sometimes if I feel dizzy, I should go to the nearest nurse or doctor. As sometimes it could mean that you do not have enough blood. (IDI-Inf. 23)

#### Acquiring knowledge about the importance of birth preparedness

Women who had given birth at the hospital reported that they were influenced by their mothers and grandmothers to refrain from preparing ahead, to avoid miscarriage. Meanwhile, the midwives scolded them for having presented themselves at the hospital unprepared. The participants acknowledged that Mobile Midwife relieved them of this predicament by assisting them in reasoning logically, to save some money to buy items that were needed for the birth.

If I go into labor today and I give birth, maybe I might not have anything and during that time I might not have any money to go and buy anything. So when I am 1 month pregnant I can begin to buy things like powder, by the time I am two months I would have added a bar of soap and by the time I am giving birth I would have gotten almost all the things I need before the baby is born. (FGI 2)

Additionally, the women were enlightened about the importance of being equipped with transportation arrangements in order to avoid any further delays during labor – for example, having the telephone number of a driver who is nearby in case of emergency. When making comparisons between Mobile Midwife's services and the ordinary antenatal services, the opinion voiced was that Mobile Midwife complemented areas that were limited at the antenatal clinic.

When the Mobile Midwife was not there, it would take a long time before going to the hospital and getting information but with the Mobile Midwife it gives information every week. (FGI 1)

### Gaining the self-confidence to care for their children

Grandmothers, who also happened to be TBAs, played an influential role not only during pregnancy, but also the postpartum period. The women depended solely on newborn care traditions passed on from their grandmothers, which at times posed a risk to the health of the child.

When the child is born and they finish wiping him, our grandparents would get the white part of bread and soak it in water and give it to the baby; sometimes the baby would be made to eat things that made them sick. (IDI-Inf. 3)

This put the women in a situation where they were desperate for other ways of caring for the child. Learning about more healthy newborn care practices from Mobile Midwife empowered the women. They were equipped with the necessary knowledge and tools needed to make them feel self-confident and trusting in their own abilities to take ownership in caring for their newborn.

I have had a lot of benefits from it. They [Mobile Midwife] have taught me the kinds of things whereby I can keep my baby clean and healthy such that even when someone sees the baby, they would want to hold him. (IDI-Inf. 27)

#### Adopting new ideas on how to care for babies’ well-being

The women used local herbs to heal the newborn's umbilical cord, sometimes leading to infections. It was also common for their babies to have very high body temperatures when teething, leaving the new mothers very worried and not knowing how to manage the situation. The informants spoke about their education on different aspects concerning care of the newborn.

At the time he was teething, his body temperature went high so the Mobile Midwife taught us that we should wet a towel with cold water and use it where … you see when the teeth are about to come out there is a small white part on the gum so we should use the towel and rub it there. They taught us all this. At first the baby would cry all the time and we would just hush them. [Laughter] (Inf. 17)

#### Learning how to ensure optimal feeding

Exclusive breastfeeding and the introduction of basic foods to the infant were matters of great concern. Had it not been for Mobile Midwife's advice, the nursing mothers would have continued to give their newborns water and inappropriate foods directly after birth. The new mothers were taught the essence of exclusive breastfeeding, and mothers who had nursed children before understood how healthy their recent babies were, compared to those who were not exclusively breastfed.

I can tell the difference in strength and health between my firstborn whom I gave water and food before 6 months of age and my second born whom I gave water and food after the first 6 months. (FGI 1)

Not knowing what kinds of foods to introduce to their babies after the period of exclusive breastfeeding was a common worry. To their relief, the mothers received suggestions in good time, about basic foods to introduce to their children. Ultimately, the women expressed that lessons learnt from the Mobile Midwife were valuable and were learnt for a lifetime.

If I was to get pregnant or give birth again and even if the Mobile Midwife did not call me again, I know the steps to take that will enable the baby to be healthy. Since they have already taught me, I have it at the back of my mind. (IDI-Inf. 23)

## Discussion

The main findings were that although they oscillated between modern and traditional practices, the women gradually gained trust in Mobile Midwife's counselling and attempted to strike a balance between myth and reality regarding nutrition in pregnancy. Moreover, their decisions to seek essential obstetric care were enhanced by Mobile Midwife's advice. Women also felt strengthened by understanding the importance of seeking professional care during pregnancy and childbirth as well as recognizing signs of ill health in the newborn.

The women gradually gained trust in Mobile Midwife's counselling and slowly moved away from traditional practices that could be detrimental to their health, like douching to cleanse the unborn baby of illness and ‘dirt’. Laura et al. (29) assessed existing beliefs and practices in maternal and child health in the same setting with the intention of tailoring Mobile Midwife's messages to meet the needs of the women. They reported that douching is at the apex of traditional medicine in the Ghanaian context; and these are performed throughout pregnancy. Douching can be done every fortnight, and as pregnancy progresses and labor approaches they can be given as often as every third day. The main concern is that some of the herbal mixtures used are quite strong and can have adverse effects on the fetus (29).

The Mobile Midwife app constantly sent the women reminders of upcoming antenatal appointments and their related importance. The women asserted that the frequency of their antenatal attendance had improved and they gave birth to their most recent babies at the health facility. Several writers (3, 12, 30) have acknowledged the potential increase in use of antenatal services in interventions where mobile phones are used. Lund et al. (11) studied the impact of a mobile phone intervention called *Wired Mothers* in Zanzibar and found that antenatal clinic attendance improved among those in the intervention group as compared to the control group.

Through Mobile Midwife, the informants appreciated the importance of avoiding malaria, especially in pregnancy. They slept in insecticide-treated nets and were consistent in their use of the malaria prophylaxis medicine SP. Brabin et al. (31) reinforces that the above-mentioned preventive measures together with intermittent preventive treatment and case management should be the focus of interventions in areas where the disease is highly endemic. Researchers (32, 33) agree that malaria is a major public health concern, especially in sub-Saharan Africa. Wilson et al. (32) claim close to 50 million women are prone to suffering from malaria every year, of which pregnant women are the most susceptible.

The women who were mainly farmers had access to foods that would serve as a balanced diet, but they did not know or understand the importance of eating foods rich in protein and vitamins. The informants also avoided eating certain fruits due to nutritional myths and therefore lost a good source of vitamins in pregnancy. Studies conducted in Kenya, Nigeria, and Iran (34–36) on nutrition in pregnancy all confirmed that nutritional health status, particularly in pregnancy, plays an important role in the health of both mother and baby. Undernutrition in pregnancy leads to low weight at birth and most likely to lifelong health risks. It is therefore vital that information regarding nutrition be provided early in pregnancy to avoid related sequelae (35). As the women began to adopt a balanced diet as recommended by Mobile Midwife, improvements in their health were reflected not only in their medical reports, but also in the health of their newborns.

Birth preparedness as well as timely health care seeking was considered to be crucial information provided by Mobile Midwife. This information helped the women to overcome the first and second phases – delay in decision to seek help and delay in decision to access health facilities – in the three delays model discussed by Combs Thorsen et al. (37) and Knight et al. (38). Kabakyenga et al. (39) further highlight that women who prepare well ahead of delivery time are more likely to be alert and make quick decisions to seek help in any cases of maternal emergency. The Mobile Midwife app also provided support for husbands, as they could subscribe and listen to the messages. Kakaire et al. (40) reported that the level of male involvement, especially in sub-Saharan Africa, is very poor, and pregnancy is viewed solely as the woman's responsibility. According to the women in our study, Mobile Midwife enhanced levels of male involvement during pregnancy, which might possibly contribute to the achievement of MDG 5. Kabakyenga et al. (39) confirm that women whose husbands were involved in deciding the place of birth were more likely to be aided by a skilled birth attendant, as compared to others.

Adherence to harmful traditional newborn care practices and neglecting to exclusively breastfeed were identified as other matters to which Mobile Midwife attended. For instance, the women used local herbs for cord care, which often led to infections. A study in Uganda revealed that Ugandan women also apply local herbs to care for the umbilical cord (41). Proper hygienic cord care has contributed to reduction of the burden of neonatal tetanus globally, from 600,000 in the 1990s to less than 60,000 in 2008 (42). Educating women residing in remote areas on newborn care through the Mobile Midwife app is a potentially powerful intervention that can further reduce neonatal deaths. With regards to exclusive breastfeeding, Thu et al. (43) reports breastfeeding benefits such as reduction in postnatal infant deaths and improvement in the child's cognitive development. This further translates into benefits in economic savings for the family, early initiation of the child into school, and thus, an incremental step into achieving MDG 2 – achieving universal primary education.

From this study we suggest that Mobile Midwife helps the target group to be aware of some possible threats to pregnancy and the newborn and at the same time helps them to overcome the potential barriers to new ideas. Mobile Midwife gives the women solutions that are easy to attain and not too far removed from their subjective realities. The free maternal health care services that were implemented at the time of the introduction of Mobile Midwife also play a significant role in enhancing the success of the Mobile Midwife. According to the women, the free antenatal clinic and childbirth services make it possible for them to save their money towards other pregnancy- and childcare-related expenses. This possibility may in turn increase the level of self-efficacy, an aspect considered as crucial in the HBM, among the women and help them fulfill the desired outcomes of Mobile Midwife (17).

The study findings verify that the success of implementation of interventions like the Mobile Midwife depends also on existing policies such as free maternal health services. Programs aiming to replicate Mobile Midwife have to be context specific and tailored towards the needs identified by the targeted groups themselves. It is not enough, however, to only secure factors that enhance self-efficacy at an individual level. Aspects relating to sustainability as indicated in the ninth Sustainable Developmental Goal (SDG), such as the building of a resilient infrastructure, promotion of inclusive and sustainable industrialization, and fostering of innovation, all have to be considered. If such technological innovations are to be successful, key players at both the national and local levels in health systems need to be empowered to take ownership of the technology and to be able to transition from grant-funding to self-supporting mHealth programs (44).

### Methodological considerations

*Credibility* was enhanced by purposive sampling of participants from different communities with varying experiences of Mobile Midwife, choosing a study setting in which Mobile Midwife was active, use of direct quotations from the interview text in the results, and assessment of the completed interviews to seek improvements in next set of data collection (26). *Transferability* was enhanced through the method of data collection, choice of study participants, and method of analysis (26), as these aspects may potentially be transferrable to similar settings provided the specific features of that context are included. The aim, specific objectives, and semi-structured interview guide enhanced the study's *dependability* (26). AAE, being a novice researcher, never having experienced motherhood, and not having a midwifery background, helped by being neutral throughout data collection, thus increasing *confirmability* (19).

### Strengths and limitations

In an effort to tailor the content of the Mobile Midwife messages to better reflect the needs of its end users, a qualitative formative study by Laura et al. (29) was conducted. The formative study reviewed a number of harmful social and cultural practices in maternal and child health (29). Apart from the study by Laura et al., little research has been conducted particularly on the Mobile Midwife program in Ghana, which renders the actual study one of very few available in this field. It is hoped that the findings of this present study will contribute to the knowledge gap concerning how integration of mHealth innovations like Mobile Midwife can lead to improvements in maternal and child health outcomes in resource-poor settings.

The FGD that had 12 participants posed a challenge to the moderator to ensure equal participation and interaction. However, according to Kitzinger (21) and Dahlgren et al. (19), it can be argued that larger focus groups allow the exploration of critical interactions and many opinions about the phenomenon under examination (20). AAE was fully aware of the influence of a male note taker on the level to which participants were able to open up in the discussions.

## Conclusions

The findings indicate that Mobile Midwife could be an excellent tool in working towards the improvement of maternal health. Mobile Midwife may hopefully contribute to the stepwise achievement of the SDGs extended from the MDGs, which expire at the end of 2015. There is need for strong political will from key stakeholders, to embark in the field of mHealth as complementary means to strengthen health systems. It is not merely the integration of mHealth activities like Mobile Midwife that will improve maternal and child health in Ghana; investments to increase numbers of skilled birth attendants are vital. Further research is needed, to unearth the ‘hidden’ challenges of programs such as Mobile Midwife in being integrated into the health systems of low-resource settings.

### Recommendations

Mobile Midwife messages about family planning informational services could be included in detail. The length of time for which Mobile Midwife messages are sent to nursing mothers could be extended from 1 to 2 years. Lastly, Mobile Midwife could include information on how HIV-positive expectant parents can take care of themselves during pregnancy, as well as their newborn.
